# Approaching Bounded Rationality: From Quantum Probability to Criticality

**DOI:** 10.3390/e23060745

**Published:** 2021-06-13

**Authors:** Lucio Tonello, Paolo Grigolini

**Affiliations:** 1 Center for Nonlinear Science, University of North Texas, P.O. Box 311427, Denton, TX 76203-1427, USA; paolo.grigolini@unt.edu; 2 GY Academy Higher Education Institution, E305, The Hub Workspace, Triq San Andrija, SGN1612 San Gwann, Malta

**Keywords:** DMM, quantum probability, conjunction fallacy, failures of commutativity, criticality

## Abstract

The bounded rationality mainstream is based on interesting experiments showing human behaviors violating classical probability (CP) laws. Quantum probability (QP) has been shown to successfully figure out such issues, supporting the hypothesis that quantum mechanics is the central fundamental pillar for brain function and cognition emergence. We discuss the decision-making model (DMM), a paradigmatic instance of criticality, which deals with bounded rationality issues in a similar way as QP, generating choices that cannot be accounted by CP. We define this approach as criticality-induced bounded rationality (CIBR). For some aspects, CIBR is even more satisfactory than QP. Our work may contribute to considering criticality as another possible fundamental pillar in order to improve the understanding of cognition and of quantum mechanics as well.

## 1. Introduction

The “bounded rationality” mainstream is based on interesting results of psychological experiments, challenging classical probability (CP) and violating its fundamental laws [1,2]. Scientific literature reports that quantum probability (QP) can correctly describe such behaviors [3], sometimes leading to the suggestion that the unique brain capabilities should rest on the law of quantum mechanics, thereby making it the fundamental pillar of the emergence of the mind [4,5,6,7]. Such a debate is still open, with controversial positions (e.g., see the commentary debate of Pothos and Busemeyer [1], and the position of Behme [8], Dzhafarov and Kujala [9], Houston and Wiesner [10]).

On the other hand, a growing body of work is focusing attention on the theory of complexity, supported by evidence of critical dynamics measured in the brain [11].

A paradigmatic model employed to study criticality is the decision-making model (DMM) [12]. The DMM has been employed as a possible model to mimic brain behavior. We have tried to investigate whether the DMM can deal with bounded rationality issues at least as much as QP, beyond the limit of CP.

In Section 2, we introduce the DMM with some insights into interesting features emerging from computational simulation. In Section 3, two fundamental bounded rationality issues (the “Linda issue” and the “Gallup poll”) are discussed from the DMM perspective. Section 4 is devoted to the final discussion and conclusion. Simulation details are presented in Appendix A and Appendix B.

## 2. The Decision-Making Model and Committed Minority

### 2.1. The DMM on a Lattice

A paradigmatic model employed to study complexity and criticality is the decision-making model (DMM) [12]. The DMM belongs to the universality class of kinetic Ising models, as discussed by Turalska and West [13], in accordance with the hypothesized arguments by Grinstein et al. [14]. The DMM approach could resemble unsupervised artificial neural networks (ANNs). Indeed, beyond some similar aspects, the unique roots of a DMM should be noted, deeply based on the concept of complexity and criticality, whereas an unsupervised ANN, e.g., the self-organizing map, is based on methods such as linear vector quantization, and hence on a proper distance measure within its own lattice [15].

**Figure 1:**
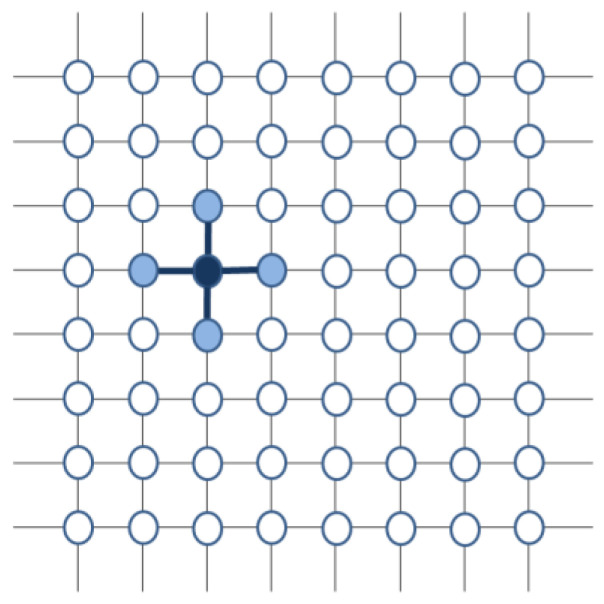
The DMM lattice. A part of a DMM lattice where the possible interactions of a node are shown with thicker lines.

In one of its proposed configurations, the DMM employs a network of N elements located in a 2-dimensional square lattice. Each element (a “node” of this network) interacts with its nearest neighbors, usually four (up, right, down and left) (Figure 1).

More precisely, each element is a stochastic node described by *s (i,t)*, a dichotomous function, taking into account that each individual can be in one of two possible states only, either +1 or −1. At each time step *t* (i.e., iteration), each node of the lattice is updated according to master equations based on the transition rates *g_i_*:

(1) $$g_{i}\left(1\to 2\right)=g_{0}exp\left[-\frac{K}{M}\left(M_{1}-M_{2}\right)\right],$$

(2) $$g_{i}\left(2\to 1\right)=g_{0}exp\left[\frac{K}{M}\left(M_{1}-M_{2}\right)\right],$$

where:

*g*_0_ = constant value;
*M* = the total number of nearest neighbors to individual *i* (e.g., four);
*M*_1_ = the number of nearest neighbors in state +1;
*M*_2_ = the number of nearest neighbors in state −1;
*K* = coupling constant (or control parameter).

The dynamics of the single node at each time step are easily described as follows: if a node *i* is in state = +1, then its probability to move to state = −1 is given by $g_{i}\left(1\to 2\right)$; if a node *i* is in state = −1, then its probability to move to state = +1 is given by $g_{i}\left(2\to 1\right)$.

In order to evaluate the global state of the network, the model defines the *global order variable* as follows:

(3) $$\xi \left(K,t\right)=\frac{1}{N}\sum_{i=1}^{N}s\left(i,t\right),$$

**Figure 2:**
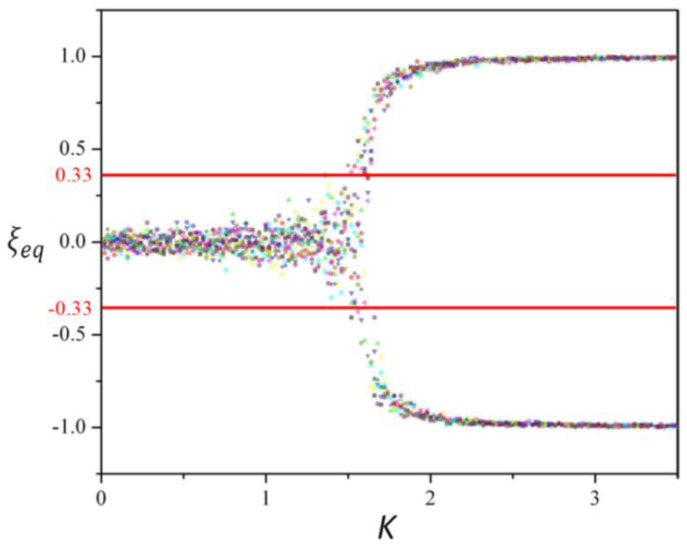
As a qualitative example of $\xi_{eq}$ versus *K*, a periodic square lattice case with N = 1024, 32 by 32 is shown; two red lines are superimposed (at +0.33 and −0.33) in order to denote the threshold for $\xi_{M}$. It should be noted that before the critical value (*K* = 1.5 for this particular DMM configuration), the fluctuations are within the region with the two straight lines as borders. At *K* = 1.5, it is evident that the fluctuations reach the borders for slightly larger values of *K*, and begin to go beyond them (adapted from [16], published according to IOP Copyright policy).

$\xi \left(K,t\right)$ is the mean value of all of the elements, hence it is not dichotomous but can span in the [−1; +1] interval. Interestingly, $\xi \left(K,t\right)$ depends on the value of the coupling constant *K*. If *K* = 0, all of the nodes in the lattice are independent Poisson processes since $g_{i}\left(1\to 2\right)=g_{i}\left(2\to 1\right)=g_{0}$, thereby making $\xi \left(K,t\right)$ vanish for a very large N. As *K >* 0 increases, network elements become more and more dependent on one another, moving away from the zero average. When *K* reaches the value *K = K_C_*, the “critical value,” a kind of “phase transition” to a global majority state, occurs. This global majority is described through the value $\xi_{eq}$ expressing the mean value of $\xi \left(K,t\right)$ over a proper number of iterations (e.g., for a fixed *K*, the mean value is taken over 10^6^ iterations), so that $\xi_{eq}=\langle \left|\xi \left(t\right)\right|\rangle$ (see Figure 2).

The $\xi_{eq}$ outcome is a real number. In order to express the population majority in brief, we introduce the variable $\xi_{M}$, defined as follows:

$\xi_{eq}\;$< − 0.33                    then $\xi_{M}$ = −1;
−0.33 ≤$\;\xi_{eq}\;$≤+ 0.33        then $\xi_{M}$ = 0;
$\xi_{eq}\;$> 0.33                       then $\xi_{M}$ = +1.

The interesting behavior is that the network shifts from a configuration dominated by randomness to an organized state, as *K* is larger than *K_C_*. In fact, for a subcritical coupling constant (*K* < *K_C_*), the single element is weakly influenced by its neighbors and changes its state with a rate close to *g*_0_ such that the $\xi \left(K,t\right)$ shows a small amplitude but very fast fluctuations around the $\xi \left(K,t\right)=0$ axis ($\xi_{M}$ = 0). On the other hand, for the supercritical coupling constant (*K* > *K_C_*), the strong interaction among elements leads to a majority state in which almost all of the elements agree to adopt the same state (+1 or −1) and stay in that “stable” state ($\xi_{M}$ = −1 or $\xi_{M}$ = +1). The coupling constant *K* is a kind of control parameter whose critical value *K_C_* corresponds to a phase change. In particular, when *K* is close to *K_C_*, the network is at criticality and $\xi \left(K,t\right)$ fluctuates, alternating its stable states +1 and −1 in a square wave fashion. In particular, $\xi \left(K,t\right)$ either stays at +1 and −1 or crosses the $\xi \left(K,t\right)=0$ axis, with times intervals following an inverse power law.

Moreover, it has also been shown that at a critical state, the network has other notable properties [12]; in particular, it has the highest measure of spatial influence (measured through the correlation function), i.e., the highest long-range interaction capability.

### 2.2. The DMM and Committed Minorities

The DMM dynamics become very interesting when there are committed minorities, namely, when a small group of elements stays in the same fixed state (+1 or −1), regardless of the choice made by their nearest neighbors. They are also called “zealots” [17]. The authors of [17] propose a theory where the individuals of a given network make decisions on whether to adopt cooperation or defection through the DMM and select the control parameter *K* so as to fit their payoff. The system makes a spontaneous transition to the critical value of *K* and the zealots exert a deep influence, benefitting from the properties of criticality that we use in this paper for the main goal of exploring bounded rationality as an effect of criticality.

We note that when the lattice is in a subcritical condition, committed minorities have no effect on the DMM lattice. In fact, the *K* parameter (coupling constant) is too small and the influence between different nodes is too weak, even the influence exerted by committed nodes. The influence of committed minorities is also negligible when the lattice is in a supercritical condition. After the occurrence of a phase transition, the network is in a definite state (+1 or −1), and it stays in that state and a committed minority cannot move it away because *K* is too strong, and the majority’s decision lasts.

Notably, when a system is at criticality, a very small group can drive the whole system to a definite state. A committed minority, usually a few nodes on the lattice keeping a fixed decision of either “yes” or “no,” either +1 or −1, can influence all of the population, independently of the other elements’ free opinion. Interestingly, under proper experimental conditions, a minority of just 1% can force the whole network to adopt its opinion (state). It can be shown (see the Appendix A) that when there are no committed minorities, the DMM lattice alternates its stable states (it alternates $\xi_{M}$ = −1 and $\xi_{M}$ = +1 in a square wave fashion). On the other hand, when there is a 1% committed minority, whose nodes are fixed in one state (e.g., +1), this minority can drive the whole DMM lattice to the same stable state (in our example, $\xi_{M}$ = +1). The whole network stays there (without moving to $\xi_{M}$ = −1) as long as the committed minority remains in action. For example, in Appendix A, a committed minority of four nodes (e.g., fixed at +1) can drive a 400-node DMM lattice (20 × 20) to a stable state (in the example, $\xi_{M}$ = +1).

**Figure 3:**
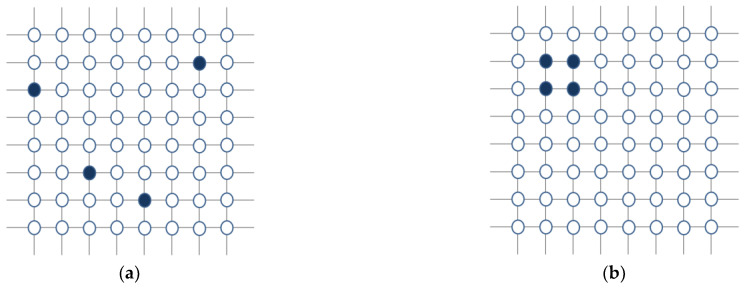
Committed minority configurations: (**a**) an example of “sparse config”: each fixed node (in dark) is located in a random position; (**b**) the “compact config”: fixed nodes form a unique square lattice.

We investigated the influence on the system of two different kinds of committed minorities at criticality. We define “sparse config” as when each committed node is randomly distributed through the lattice and “compact config” as when the committed nodes act as a unique square lattice, i.e., four nodes corresponding to a 2 × 2 square lattice (see Figure 3).

Appendix A shows that when only one committed minority is present, it drives the DMM lattice to the related state, regardless of whether it is sparse or compact.

Appendix A shows that, as expected, when both sparse and committed minorities share the same opinion, the outcomes are not interesting. If both minorities are fixed at +1, then $\xi_{M}$ = 1, and if both are fixed at −1, then $\xi_{M}$ = −1. If they do not share the same opinion, but both are sparse or compact, then $\xi_{M}$ = 0.

Interesting outcomes appear when sparse configurations versus compact configurations act at the same time, with conflicting opinions. When different configurations challenge each other, the results of Appendix A show that:

Sparse config = −1 versus compact config = +1 yield $\xi_{M}$= −1

Sparse config = +1 versus compact config = −1 yield $\xi_{M}$= +1

In brief, the sparse config somehow “dominates” the compact config, exerting a stronger influence on the population dynamics. This is a very important result.

It is easy to understand these results. In the sparse configurations, each committed node influences four neighbors, thereby making the committed minorities influence 16 nearest neighbors, while in the compact configuration, the number of nearest neighbors is just eight, hence exerting weaker influence on the system (see Figure 3).

We make the interpretation of these results more interesting by adopting the perspective of lookout birds [18]. According to the authors of [17], the DMM lattice is interpreted as a flock of birds (each node is a bird). Each bird has to make a choice between flying right (+1) or left (−1). When *K* is subcritical, the decision rate is given by *g*_0_ and there is no cooperation. When *K* is supercritical, the flock stays with its decision, with no significant change at all. However, when *K* is at criticality, we use the comparison metaphor to explain the influence of lookout birds on the decision made by the flock. As stated earlier, the lookout birds are either compact or sparse. If they are sparse, they perceive a wider target, namely, they have a better understanding of the environment. They may perceive either a more abundant source of food, exerting an influence on the flock to move toward it, or a more dangerous predator, thereby exerting an influence on the flock to move far away from it. The compact lookout birds, due to their smaller angle view, may perceive fewer resources than the sparse lookout birds. The flock makes a comparison between the two conflicting choices and decides to either move towards the more abundant source of food or to move far away from the more dangerous predator.

The metaphor of sparse lookout birds can be used not only to realize a faster information transmission, adopting the information paradigm, but also an improved computational capability, adopting the computational paradigm. In fact, the swarm (or flock) can be viewed as a device where the committed minorities (either sparse or compact) are the inputs and the global value $\xi_{M}$ is the output of an operation, namely, a comparison.

### 2.3. The Model

**Figure 4:**
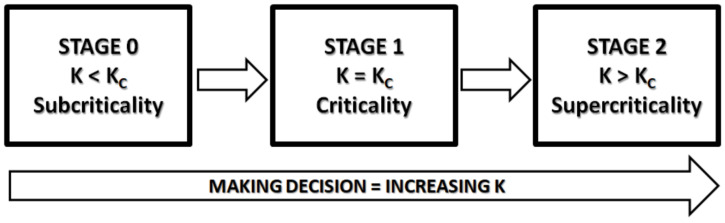
The proposed steps of how a DMM makes a “decision”.

The well-known Ising model describes a phase transition, moving the system with a control parameter (*K*) from a subcritical, to a critical and, next, to a supercritical stage (or vice versa). Now, let us consider the DMM. Let us suppose the same dynamics describing, in a sense, a phase transition: *K* moves the DMM from subcriticality (stage 0) to criticality (stage 1) and, next, to supercriticality (stage 2). This latter stage is a sort of collapse, reminiscent of quantum mechanical wave function collapse, representing the decision made by the DMM system (see Figure 4).

- At “stage 0,” the lattice is at subcriticality (the order parameter *K* < *K_C_*) where committed minorities have no effect.
- At “stage 1,” *K* increases to *K_C_* and the lattice is at criticality, where committed minorities can operate (in a type of superposed state) and drive the lattice to a definite configuration, or at best, to a global state $\xi_{eq}$, of course characterized by a definite number of nodes in state 1 and others in state −1.
- In “stage 2,” *K* is moved to supercriticality (*K* > *K_C_*) so that the lattice obtained from criticality can somehow “collapse” to one of the possible states (only +1 or −1 states are allowed).

As stated earlier, at stage 0, committed minorities have no effect. At criticality (stage 1), the choices made by the committed minorities are not yet the system’s decision: they are the source of a sort of superposition of states. Only at stage 2 does the system collapse to a definite state. In conclusion, within the DMM perspective, making a decision means increasing *K*, as in a phase transition.

Now, let us consider the supercritical stage only (stage 2). As the DMM goes to *K* > *K_C_*, it moves to a definite equilibrium state (+1 or −1 only). In Appendix A, we show that at supercriticality, a DMM collapses to a definite state (+1 or −1) according to the majority of the states expressed by its elements: if its nodes at +1 are more than 50%, then the DMM goes to +1; if its nodes at −1 are more than 50%, then it goes to −1. Therefore, the final layout at stage 2 depends on the layout of the DMM at the beginning of stage 2, which is the same as at the end of stage 1. Committed minorities can influence the structure of this layout.

Let us consider the lattice at stage 1 (at criticality) and its layout. Although its mean value is given by $\xi_{eq}$, it changes in any iteration, slightly or extensively. Therefore, the subsequent and final “collapsed state” at the supercritical stage will depend on the particular iteration when the lattice at criticality is considered as the starting point for stage 2.

Let us consider the lattice at criticality (stage 1) and an interval of 10^6^ iterations (this magnitude order is in line with simulation standards of [12]): it corresponds to 10^6^ possibly different lattice output layouts. According to the dynamics described above (see details in Appendix A), a good approximation is realized as follows: for each iteration at stage 1, if its layout is $\xi_{eq}\;$*>* 0 (more +1 than −1 nodes within the lattice), the supercritical lattice will “collapse” to a +1 stable state, while when $\xi_{eq\;}$< 0, the supercritical lattice will “collapse” to −1.

**Figure 5:**
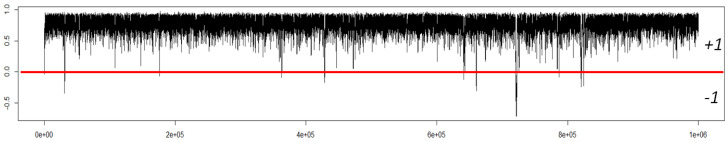
The graph shows the $\xi_{eq}\;\mathrm{value}$ of a DMM (10^6^ iteration) at criticality with a sparse committed minority set at +1. In particular, it can be seen that only in a few cases (0.2%) is the layout $\xi_{eq}\;$ < 0, thus leading in step 2 to a −1 state. Hence, for almost all of the cases, the system will collapse to a +1 state.

The dynamics can now be expressed in terms of probability. As an example, consider a lattice at criticality (stage 1) with a sparse minority at +1. Now, let us look at its 10^6^ layouts. In a real simulation of 10^6^ iterations, we found that only n = 1805 layouts (about 0.2%) were $\xi_{eq}\;$*<* 0. This result leads us to state that the probability that a sparse minority at +1 drives the supercritical system to +1 is *p* = 0.998, namely, the system collapses to the +1 state 99.8% of the time (see Figure 5). It is like a “phase transition” where there are two possible alternatives (e.g., think of magnetization in an Ising model) and they are chosen probabilistically.

We simulated three cases at criticality in order to evaluate the probability of having $\xi_{eq}\;$< 0 for each of them:

1. A compact committed minority set at −1,
2. A sparse committed minority set at +1,
3. A compact committed minority set at −1 and a sparse committed minority set at +1.

Each case is simulated starting from a random DMM layout and the first 10^6^ iterations are considered. The number of nodes selecting +1 or −1 over the 10^6^ steps allows us to evaluate their percentage over time. For each of the three cases, this evaluation is repeated 10 times and mean values (over this 10 times) are reported in Table 1. In particular, Table 1 reports data on the number of nodes selecting −1 (i.e., the case $\xi_{eq}\;$< 0).

Using probability arguments, we conclude that a DMM with a compact minority in the state −1 has the probability 0.956 of driving the majority of its elements to the same state. Moving to the supercritical condition has the effect of setting the whole lattice to the definite −1 state. In other words, moving from a random initial layout with compact committed minority established in the subcritical condition to the critical condition and finally to the supercritical condition has the effect of making the system collapse to the same state as the committed minority with probability *p* = 0.956. Note that a sparse minority set at +1 has probability *p* = 0.003 of leading to a −1 state while a compact (at −1) vs. a sparse minority (+1) has probability *p* = 0.043 (note, one order of magnitude greater than the sparse minority only) of leading to a −1 state.

We adopt the following interesting interpretation of the results of the numerical simulation. The stage 1 is the step at which the system “compares” (calculates, processes information) and makes a decision which becomes “real” with a clear, definite and known probability, in the supercritical state (i.e., in stage 2).

Stage 1 (criticality) can be viewed as a sort of coherent, superposed quantum state; think of 10^6^ iterations where the DMM layout changes continuously (like a sort of wave function) but with a probability of having a definite $\xi_{eq}$ or even with a probability that $\xi_{eq}$ would be in a definite range, e.g., ($\xi_{eq}<0$ or >0) somehow as the probability of an electron and an orbital.

Stage 2 (supercriticality) is interpreted as a sort of “wave function collapse,” a form of “quantum decoherence”; once in this stage, the lattice is set in a “stable” definite state in which it stays. In other words, the dynamics of a DMM phase change yields interesting properties, usually described by quantum mechanics and quantum probability. In a sense, the DMM dynamics resemble quantum coherence, in line with [19]: “Quantum coherence refers to the ability of a quantum state to maintain its entanglement and superposition in the face of interactions and the effects of thermalization”.

Let us stress an interesting similarity with quantum mechanics. When both minorities, sparse and compact, are set in a DMM at criticality, the cloud of fluctuations is asymmetrical with a negative component more intense than the positive one (see Table 1). This is a property similar to quantum mechanics generating a superposition of two states, with different expansion coefficients. If we increase *K*, we generate a collapse of the wave function and this collapse yields the state |−1> with a higher probability than the state |1>.

Of course, there is a significant difference between quantum and complex perspectives, but this difference is worthy of discussion. The DMM “superposition of states” does not set any limit on our knowledge. We know any iteration, we can see it, we can “handle” it, whereas in quantum mechanics, the coherent superposed state cannot be observed at all. We state that the DMM superposition describes a kind of “transparent” (non-quantum) coherence.

### 2.4. The Model: A Graphic Representation

**Figure 6:**
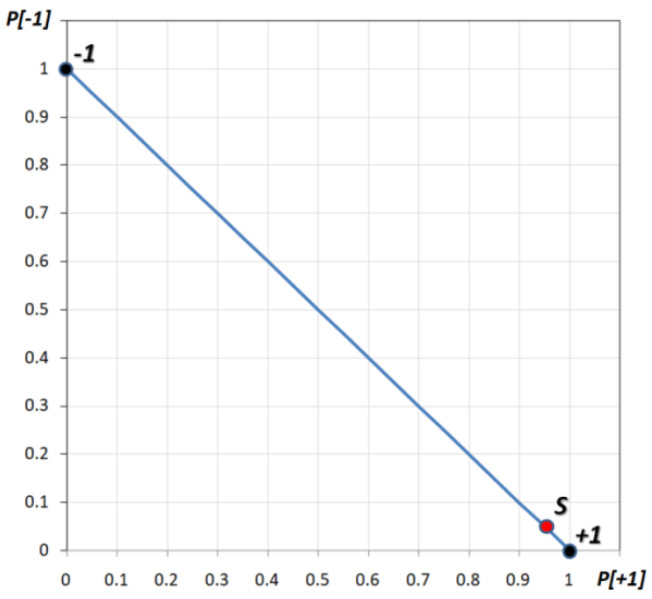
The graph where in the horizontal axis shows the “+1” outcome probability while the vertical one shows the “−1” outcome probability. The outcome of sparse and compact committed minorities is expressed with the point S (red dot on graph), where 4.29% of “−1” outcome can be read on the vertical axis while, of course, 95.71% of the “+1” outcome can be seen on the horizontal axis. The blue line represents the possible probabilities of stage 1 while the two extreme points (+1 and −1, see figure) are the 2 possible “stable” states in supercritical conditions.

We can represent this model with a graph in which the horizontal axis represents the “+1” outcome probability while the vertical one represents the “−1” outcome probability (see Figure 6). Therefore, e.g., the outcome of “sparse and compact committed minorities” (case 3 of the above) can be expressed with the point S (red dot on the graph), where 4.29% of the “−1” outcome can be read in the vertical axis while, of course, 95.71% of the +1 outcome can be seen in the horizontal axis. Please note the blue line is the locus of all possible probabilities whose extremes are −1 and +1 which correspond to *p* = 1.0 of being at status −1 and +1. Once the system goes towards supercriticality, the points must move towards the −1 or the +1 point, therefore collapsing to a “definite” state.

**Figure 7:**
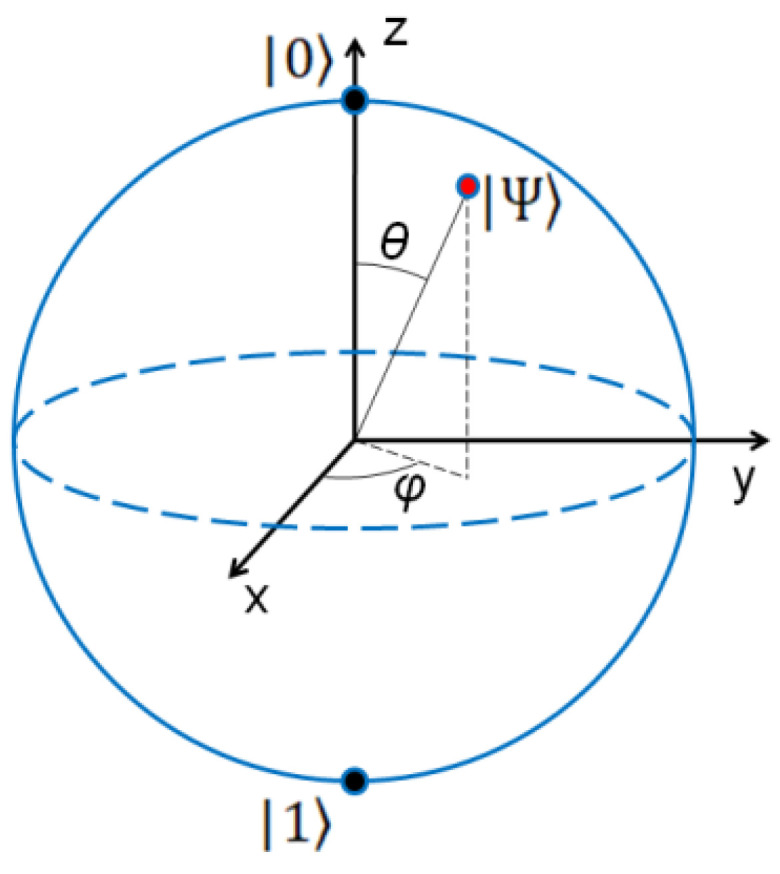
The Bloch sphere as a qubit graphic representation.

The blue line represents the possible probabilities of stage 1 while the two extreme points (+1 and −1, see picture above) are the two possible “stable” states in supercritical conditions towards which the system must move. This resembles the description of the Bloch sphere (Figure 7) for a qubit: the sphere surface represents all of the possible superposed states (as does the blue line in our graph) while the two extremes |0> and |1>, the “North Pole” and the “South Pole” (as the two extremes of the blue line), are the classical bits where a qubit must collapse (e.g., see [20]).

## 3. Bounded Rationality Issues

### 3.1. The “Linda” Issue

Bounded rationality is a wide and deeply studied field of research. Tversky and Kahneman worked on many of its aspects. In particular, they [21,22] defined and described the so-called “conjunction fallacy” through interesting experiments. The most studied is perhaps the “Linda issue.” In their work, they submitted to a group of subjects personality sketches of a woman called Linda followed by a set of possible occupations or avocations.

This was the description “L” of Linda: Linda is 31 years old, single, outspoken and very bright. She majored in philosophy. As a student, she was deeply concerned with issues of discrimination and social justice, and also participated in anti-nuclear demonstrations. Three of the proposed occupations/avocations were:

1. Linda is active in the feminist movement (F),
2. Linda is a bank teller (T),
3. Linda is active in the feminist movement and a bank teller (F & BT).

As reported by Tversky and Kahneman, “the description of Linda was constructed to be representative of an active feminist (F) and unrepresentative of a bank teller (BT)” [22]. In fact, the result reported that the most probable description order (85% of the population investigated) is: F > F & BT > BT.

Interestingly, beyond the confirmation of F > BT, it is impressive that the great majority of subjects also rank the conjunctions (F & BT) as more probable than their less representative constituent (BT). This is a non-rational behavior within the theory of decision making because it violates the classical probability (CP), according to which the probability of a conjunction (P [F & BT]) cannot exceed the probabilities of its constituents (P [BT]). Tversky and Kahneman named this phenomenon the “conjunction fallacy.”

A strong body of scientific literature made the proposal of using quantum probability (QP) to explain the results of this experiment [1,7,23,24], showing that QP can account for P [F] > P [F&BT] > P [BT] where CP cannot.

### 3.2. The DMM and the “Linda” Issue

Let us go back to the DMM and let us consider the “concept” of Linda as a feminist (F) as a sparse committed minority at “+1” and the “concept” of Linda as a bank teller (BT) as a compact committed minority at “−1.” Of course, the case of Linda as F & BT occurs when both committed minorities are active. In a sense, such a view is similar to the one proposed in the QP models where the “concepts” of F and BT were considered as vectors on multidimensional subspaces [1]. Using the DMM perspective, we can use the same vector representation to express the intensity of the opinions of committed minorities (we take “sparse” and “compact” just as two paradigmatic examples). In the same way, note that the concept of “F & BT” is a superposition: a vector superposition in the QP perspective, and a DMM minority superposition within our criticality perspective.

The “lookout birds” image affords another attractive interpretation based on looking at the “concepts” of “F” and “BT” as indications of a source of food for the flock. Let us interpret the “F” concept as a large source of food to the right of the flock: a sparse minority sees it and says “turn right” (namely, set the state to +1), thereby making the flock turn accordingly. Let us interpret the “BT” concept as a small source of food to the left, and a compact minority sees it and says “turn left” (namely, set the state to +1). Let us consider the case where both sources of food are present, with the sparse minority perceiving the large one and the compact minority perceiving the small one. According to the results of the numerical simulation, the flock will turn in a direction between “left” and “right.” Think of the direction of the flock as $\xi_{eq\;}$, i.e., the global DMM status (see Appendix A). In the case of a sparse committed minority only, it is a value next to 1, in the case of a compact minority, it is a value next to −1 and when both the committed minorities are present, it is a value between the two extremes.

**Figure 8:**
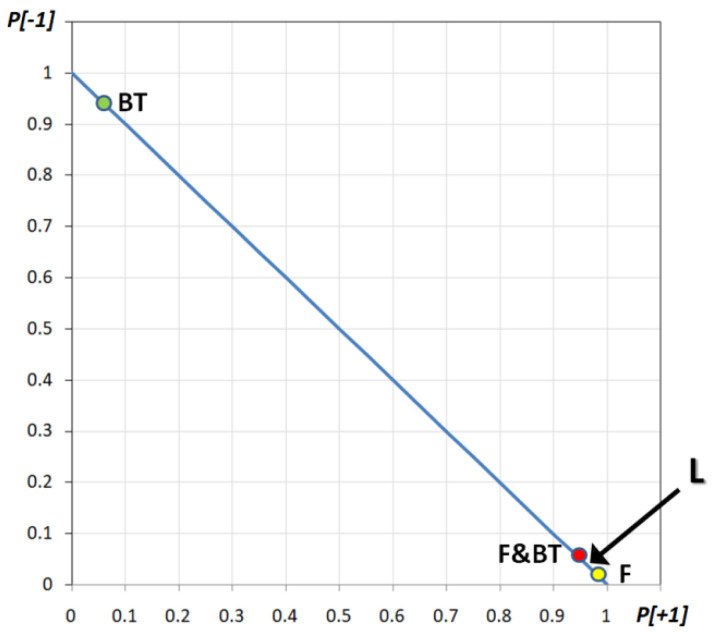
Graphical representation of DMM critical dynamics of Linda as a feminist (point F), Linda as a bank teller (point BT) and Linda as both of them (point F & BT). The area in which the full description of Linda (L) should occur is shown with the arrow L.

Let us go back to the DMM and consider it in terms of probability. The DMM at criticality, with the “pure” concept of “F” (with a sparse committed minority at +1), will dynamically activate all of its lattice nodes (at +1). Most of the time (i.e., 99.7% of the time—see the previous section and Appendix A), the majority of the nodes will be “+1” so that once the system moves to a supercritical stage, its final global state variable will be fixed at +1 with probability *p* = 0.997. On the other hand, facing the “pure” concept of BT (with a compact minority at −1), the majority of DMM nodes will be in the state “−1” 95.60% of the time, so that in a supercritical state, the final global variable will be −1 with a probability of 95.60%. When both committed minorities are present (F & BT), the DMM will yield “+1” 95.71% of the time (going to the supercritical state, the final global variable will be +1 with a probability of 95.71%). The three cases are reported in Figure 8 as point F, BT and F & BT and it should be noted that the F & BT concept has a probability between F and BT.

It should be noted that this superposition (F & BT) leads the DMM to a “dynamic state” which can occur at criticality only (it cannot be seen in a supercritical state). In fact, in the supercritical state, the DMM will always collapse to +1 or −1 because F & BT is not a “pure” dynamical state but a superposition of states. This is in line with quantum cognitive models (e.g., see [1]). A possible interpretation is that in the brain, the concept of F and the concept of BT occur. Of course, one subject can think about both of them at the same time, but it is a superposition of known concepts and not a new one, so it seems hard to think of F & BT as a stable cognitive supercritical state. F & BT could be seen as a kind of “artifact” (maybe as a bi-stable figure: one can deal with it knowing that it is made up of two figures, but one can “see” just one of them at a time). Note that, besides disappearing at a supercritical stage, at criticality, it behaves as a dynamic state in the sense that it is related to its own particular DMM dynamics, just as F and BT are. It seems noteworthy that the superposed state F & BT “lives” at criticality only, just as in the QP model [1] it lives as a coherent state.

Now consider a 4th dynamical state and let us call it Linda (L). Note that that L is a brand new dynamical state, and at criticality, it behaves just as F, BT and F & BT (L is related to its own particular DMM dynamics). Therefore, the DMM, coping with L, will activate a dynamical process of the same kind as that activated to deal with F, BT and F & BT. As a consequence, the system at criticality will spend a certain percentage of its time on the value +1 and −1, referring to L, and in the supercritical regime it will collapse with the corresponding probabilities, as we have discussed for the other states.

Tversky and Kahneman [21,22] described the new state (L) as different from F, BT or F & BT and, in their experiment, asked people to order the probabilities that L is like F, BT or F & BT. This is a fundamental point: they compared L versus F, BT and F & BT (they did not compare F, BT and F & BT!). The formal question is to compare the probability of similarity between L and F, versus L and BT and versus L and F & BT. Stating P(F) > P(F & BT) > P (BT) could be misleading because they did not find that. Instead, they found that, according to our formalism: |P(L) − P(F)| > |P (L) − P(F&BT)|> |P (L) − P(BT)|. In Kahneman’s words [2]: “… the judgments that subjects made about the … Linda problem substituted the more accessible attribute of similarity (representativeness) for the required target attribute of probability.”.

Therefore, what we have to do is evaluate the distance between L and F, BT and F & BT. In particular, Kahneman and Tversky found that almost all the subjects said Linda is F; indeed, they built the experiment so that Linda the feminist is the description that best fits the description given. Therefore, it is very likely that the probability describing L is a point very close to the point F (see Figure 8). Given that, it could be easily understood that the second closest point is F & BT, not BT, in line with the well-known “conjunction fallacy.” In fact, the distance |L − F| is very short, thus the second closer state must be F & BT and finally BT, in third place. Therefore, our model is clearly in line with the experimental result of the “conjunction fallacy.” Moreover, such an approach seems to evaluate the closeness between the state from the initial information and the state from the various questions, in a way analogous to the influential and well-known “representativeness heuristic,” suggested by Kahneman and Tversky [25].

It should be noted that this comparison occurs at criticality because any decision (or comparison or evaluation) in DMM occurs at the criticality stage only, as shown in the previous section. We do not make a comparison between definite states, because they are properties of the supercritical condition. These properties are outcomes, and we aim at evaluating the intelligent “decisions” behind them.

We try to explain these concepts with an image: suppose you are sitting on dock of a bay, looking at the sky. You are just under the route of migrating birds so you can see flocks of birds coming from the south to north again and again (i.e., year after year). You know that in the area there is a farmer seeding his fields and the birds like it as food.

When the farmer seeds food 1 in the left field (field F), the flock turns left 99.7% of the time. 

When the farmer seeds food 2 in the right field (field BT), the flock turns right 95.6% of the time.

When the farmer seeds both of the fields (fields F & BT), the flock turns left 95.71% of the time (because field 1 is larger or maybe they prefer food 1).

Now, in the new season, you can see the flock’s directions (e.g., turn left/right percentage) but you do not know what fields and foods have been chosen by the farmer. Suppose the flock turns left in new percentage, e.g., 98.0% (let us call this behavior “L”), what are the probabilities that the flock has seen field F, F & BT or BT? If you list them, of course you will find the same order of probabilities.

The Linda description (L state), being based on dynamics at criticality, is very close to the F state. As a consequence, the second closest state must be F & BT.

The described approach to bounded rationality, involving a DMM and having criticality as a core element, leads us to introduce the term criticality-induced bounded rationality (CIBR) to identify it.

### 3.3. Superposition in the “Linda” Issue

The “bounded rationality” outcome found by Tversky and Kahneman [21,22] can be described with QP or with CIBR and not with CP. The main issue is the experimental result that P [F & BT] is more probable than the single probability P [BT], in conflict with the theoretical laws of CP.

The reason why QP [1] yields an interpretation of F & BT fitting the outcomes of psychological experiments is that QP allows us to interpret F & BT as a superposition of two different states rather than as a classical sum. CIBR yields the same benefit as QP of making F and BT live together in one dynamical superposition of states, bypassing the limits of CP. This is reminiscent of QP with a noteworthy difference.

In fact, the superposed state of F and BT can be clearly seen in the lattice of a DMM so that a clearly definite probability is observed and evaluated in space and time. Instead, in QP models [1], BT and F are described only as separated projections: their superposed state cannot be observed (of course, because it is a quantum superposition, so to speak, a model of coherent state) and must be described through conjectures. Pothos and Busemeyer write: “As it is impossible to evaluate incompatible questions concurrently, quantum conjunction has to be defined in a sequential way, …that is, we have Prob(F ∧ then BT) = Prob(F)·Prob(BT|F)” [1]. In other words, the QP model cannot directly compare the case F & BT (which is a superposed state) because it has to be considered as a sequence of “collapsed” states of F and BT. Moreover, in order to evaluate this in a sequential way, in QP, other assumptions must be made, and the same authors write “an additional assumption is made that in situations such as this, the more probable possible outcome is evaluated first.” On the contrary, the CIBR superposition of the states F and BT corresponds to a well-defined cluster of decisions made at criticality.

QP is based on the superposition of F and BT, a condition inaccessible to observation if the wave function collapse is not involved. We note that the wave function collapse is still an open problem for quantum mechanics. The adoption of CIBR allows us to use both quantum mechanical concept of state superposition, at criticality, with no limits on observation, and the counterpart of wave function collapse, in the supercritical condition.

In other words, in QP, one can deal with a collapsed state only (F and BT) and the superposed state is inaccessible, whereas with the CIBR approach, one can work with a collapsed state (e.g., in the supercritical stage) as well as with the superposed state (e.g., in the critical stage). If one considers a superposed state in QM, e.g., a qubit, it could have a value of *α* and *β* as a point in the Bloch sphere, but one will never know where it is in a definite moment, while on a DMM lattice, one can always know where the point is; only in CIBR can one see what happens in the decision process, in space and time. Note that CIBR does not necessarily imply a DMM, which is just an instance. Our approach can be defined as a non-linear stochastic theory, as are the approaches mentioned by Breuer and Petruccione [26], to account for the wave function collapses in quantum mechanics.

QP and CIBR can both correctly describe the Linda issue because they seem to operate with superposition in a similar way, as opposed to the CP approach. In particular, CIBR seems to offer a better way because of its clearer approach, unlike the inaccessible quantum coherent state of QP.

### 3.4. Failures of Commutativity in Decision Making

Another interesting issue raised within the “bounded rationality” domain is the “failures of commutativity” in decision making, whereby asking the same two questions in different orders can lead to changes in responses. As an example, consider the questions “Is Clinton honest?” and “Is Gore honest?” and the same questions in a reverse order. When the first two questions were asked in a Gallup poll, the probabilities of answering “yes” for Clinton and Gore were 50% and 68%, respectively. The corresponding probabilities for asking the questions in the reverse order were 57% and 60% [27]. Such order effects are puzzling according to CP theory because they seem to violate the commutativity laws. The QP approach, as is known, can describe the experimental result, suggesting that the quantum approach accounts for the fact that thinking about one “concept” (e.g., Clinton being honest) changes the basis when the second one (e.g., Gore being honest) is evaluated.

Let us consider this issue using the CIBR perspective. If we just ask: “Is Clinton honest?”, we must consider that the complex system has to make a “decision.” Thus, the system will move from its undercritical stage to criticality when the decision is made and then it will move to a supercritical stage when the decision is fixed (the system will collapse), just a phase change as described above. It is likely that it will start from a subcritical “random” layout of nodes with, probably, *p* [+1] = 0.5 and *p* [−1] = 0.5.

On the other hand, if we make the question “Is Clinton honest?” just after asking “Is Gore honest?” the starting layout will be the one left by the previous answer (or, best, decision) or at least be influenced by it. In other words, the starting layout of the second question for CIBR could not be a “neutral” one (*p* [+1] = 0.5 and *p* [−1] = 0.5) but of course related to the outcome layout of the previous answer. For instance, suppose the first question drove the system to a supercritical “+1” state. Now, for the second question, the system goes back to the critical phase (in order to make a second decision) from the +1 supercritical branch; the critical “decision” will not start from a random layout but from a layout with, somehow, a clear and definite majority of +1 elements so it is likely to be influenced by that.

Actually, it could be argued that in the “long term” (after one hundred thousand iterations), it is expected that the CIBR with a committed minority at criticality will reach a definite $\xi_{\mathrm{eq}}$ (a definite number of +1 and −1 elements) independently from the initial condition, being mainly influenced by the committed minority. Indeed, it is also expected that, at least in the early iterations, that the story of the system would be different, depending on the starting layout.

In order to verify this, we carried out two different simulations:

Scenario A: a committed minority at −1 in a sea of +1 elements (i.e., all the elements not in the committed minority are in the state +1);

Scenario B: a committed minority at −1 in a sea of −1 elements (i.e., all the elements not in the committed minority are in the state −1).

Simulation results strongly agree with our prediction (see Appendix B): there is an initial interval that is different according to the initial starting layout so it can lead to different outcome probabilities.

Therefore, the failures of commutativity in decision making (whereby asking the same two questions in different orders can lead to changes in responses) could be due to the different CIBR dynamics. When we ask: “Is Clinton honest?”, the system at criticality leads to a layout (stage 1) which “collapses” to a definite supercritical state (stage 2). Just after that, the second question “Is Gore honest?” will move the system again to a critical state in order to make a second decision, but coming from a supercritical state where almost all elements have a definite value −1 (or +1), thus changing the outcome probability of the second question, having different outcomes at least in the first iterations.

It should be pointed out that this difference becomes evident only when there are very different initial layouts (e.g., when coming from different supercritical phases). Notably, this is consistent with the QP interpretation of different bases cited above.

## 4. Discussion and Conclusions

### 4.1. Criticality-Induced Bounded Rationality

Both the “Linda issue” and the “Gallup poll” indicate the failure of CP and both of them can be successfully addressed using QP and CIBR. Indeed, the “bounded rationality” mainstream discusses other issues and as QP is suggested as a possible unique way to describe them, so CIBR can now be suggested as well. For the latter, the two pillars seem to be the “superposition” (described to figure out the Linda issue) and the “commutativity” dynamics (described for the “Gallup poll” issue).

As an example, consider the “sure thing principle” [1]. It is the expectation that human behavior ought to conform to the law of total probability, e.g., in a one-shot prisoner’s dilemma task, participants violate the sure thing principle. In fact, usually, the player does not know the opponent’s move but when participants were told that the opponent was going to cooperate, they decided to defect; when they were told that the opponent was defecting, they decided to defect as well. The expectation from the sure thing principle is that, when no information is provided about the action of the opponent, participants should also decide to defect. However, surprisingly, in the “no knowledge” case, many participants reversed their judgment and decided to cooperate. This is named the “violation of the sure thing principle.”

In our CIBR, the knowledge of the opponent’s move could be a second committed minority (e.g., sparse) activating (e.g., at −1) and thus dominating the decision that would be made by a first committed minority (e.g., compact and set at +1) which drives the decision for collaborating. Therefore, without the knowledge of the opponent’s decision, the CIBR expresses its “natural” trend to collaborate, according to the known principle called “wishful thinking,” represented by the compact committed minority fixed at +1 but possibly dominated by a −1 sparse committed minority related to the knowledge of the opponent’s move.

In a similar way, other issues could be addressed. Tversky [28] showed that similarity judgments violate metric axioms. For example, in some cases, the similarity of A to B would not be the same as the similarity of B to A: the similarity of Korea to China was judged greater than the similarity of China to Korea. This could be viewed as a comparison between two minorities coming from the +1 or from the −1 branch of the supercritical stage, leading to different results, of course in probability terms.

### 4.2. Conclusions

Bounded rationality violates CP, making researchers suggest QP models as one of the most convincing ways to describe issues such as, e.g., the “Linda problem” or the “Gallup poll.” We have seen that this may be not a correct conclusion. The adoption of the CIBR perspective affords an alternative way of accounting for bounded rationality.

As the final remarks concerning future research work, we want to stress that to fully benefit from the adoption of the CIBR perspective, in future work, we shall adopt self-organized temporal criticality (SOTC) [29]. In fact, in the discussion of this paper related to Figure 4, we made the assumption that the control parameter *K* moves from the subcritical to the supercritical condition, crossing back and forth to the critical condition *K_C_*. The adoption of SOTC spontaneously generates that process, remaining at the level of CIBR.

This would require additional computer calculations but the comparison between CIBR and QP will not change. We are therefore inclined to draw the main conclusion that CIBR is an alternative approach to bounded rationality that for some aspects is even more satisfactory than QP. CIBR goes beyond the “uncertainty” imposed by quantum mechanics, at least in some cases. In a sense, criticality may yield a sort of non-quantum coherence, more “transparent” than quantum coherence, leading to a better understanding of cognition emergence.

The discussion about the incompleteness of quantum mechanics is out of the scope of this paper. It is still the subject of debate and we limit ourselves to mentioning a recent paper by Evans [30]. We believe that our paper proves that CIBR affords an explanation of bounded rationality that does not require quantum probability without giving up classical ontology, thereby suggesting that CIBR deserves some attention in the debate on quantum mechanical incompleteness.

## Figures and Tables

**Table 1:** Nodes selecting “−1.” The second column (named “% of −1”) reports the average value over 10 trials of the percentage of nodes selecting “−1”in a 10^6^-timestep simulation, the third column the standard deviation (SD) over the 10 trials and the fourth and fifth the minimum and the maximum results over the 10 trials. The first row (“Compact −1”) is related to a compact committed minority set at −1; the second row (“Sparse +1”) is related to a sparse committed minority set at +1; the third row (“Compact −1 vs. Sparse +1”) is related to a compact committed minority set at −1 and a sparse committed minority set at +1.

Minorities	% of −1	SD	Min	Max
Compact −1	95.60	1.97	92.09	97.67
Sparse +1	0.30	0.16	0.02	0.55
Compact −1 vs. Sparse +1	4.29	2.35	1.35	9.14

**Figure A1:**
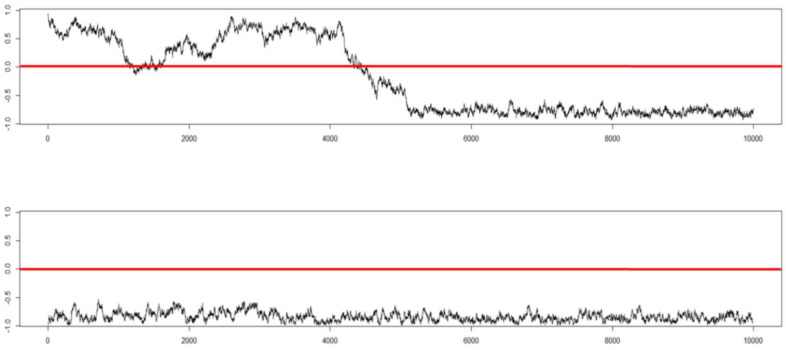
Example of a “scenario A” (above) and a “scenario B” (below). First 10^4^ iterations are shown.

## Data Availability

Data sharing is not applicable.

## Appendix A. The DMM Numerical Simulations

A DMM has been considered according to literature suggestions [11]. It features a 20 × 20 lattice of agents (total n = 400 agents) with periodic boundary condition settings and *g*_0_ = 0.1. For each simulation, in line with suggested dimensions in the literature, the total iteration number has been set to 10^6^ and the $\xi_{eq}$ has been evaluated on the last 5 × 10^5^ iterations. All simulations have been performed using R [31].

Two sets of simulations have been performed, with the first set at criticality, the second set at supercritical dynamics.

### Appendix A.1. (a) Simulations at Criticality

All simulations within this set have been considered at criticality, with *K* = 1.66. Starting conditions have been set initializing 50% of nodes at state = +1 and the other half at state = −1, with randomly selected positions (unless otherwise specified).

Three stages of simulations have been planned and performed.

In stage 1 of the simulations, the model has been firstly tested without any committed minority. Simulations confirmed what have been reported in the literature: in the critical state, without a committed minority, the global value jumps from state +1 to state −1, alternating between them without any “domination.” In particular, five simulations have been performed whose descriptive statistics were ${\bar{\xi }}_{eq}\;$= 0.09 ± 0.16 (m ± SD), clearly confirming $\xi_{M}$ = 0 when there were no committed minorities.

In stage 2 of the simulations, the model has been tested with one committed minority. As suggested in the literature [11], the committed minority has been considered as 1% of the total population, namely, just four nodes. Therefore, in stage 2, simulations have been performed fixing just four nodes at state = +1. At the first step, each fixed node has been located at a random position on the lattice, named “sparse config.” Secondly, fixed nodes have been considered as a unique 2 × 2 square lattice, with fixed state = +1, named “compact config.” The same simulations have been symmetrically performed with fixed node = −1, for a second check.

Stage two simulations confirmed that 1% of the randomly positioned committed minority move the global variable to the same value as the minority. Repeating the same simulations using a compact minority layout, the outcome is the same as the randomly positioned (sparse) fixed elements. In particular, each simulation has been performed five times (fixing state of the minorities at +1), leading to:

(a) sparse config: ${\bar{\xi }}_{eq}\;$= 0.76 ± 0.01 (m ± SD)
(b) compact config: ${\bar{\xi }}_{eq}\;$= 0.71 ± 0.01 (m ± SD)

In both cases, it is clearly confirmed that a committed minority (independently of its sparse or compact configuration) of fixed values set to +1 (or symmetrically −1) led the majority to $\xi_{M}$ = +1 (or symmetrically, −1).

In the third stage of the simulation, two minorities have been fixed in the same 20 × 20 lattice in order to see how the dynamics would evolve. All possible combinations have been evaluated, in particular:

sparse config = −1 versus sparse config = −1
sparse config = +1 versus sparse config = +1
sparse config = +1 versus sparse config = −1
sparse config = +1 versus sparse config = +1
compact config = −1 versus compact config = −1
compact config = +1 versus compact config = +1
compact config = +1 versus compact config = −1
compact config = +1 versus compact config = +1
compact config = −1 versus sparse config = −1
compact config = +1 versus sparse config = +1
compact config = +1 versus sparse config = −1
compact config = +1 versus sparse config = +1

Therefore, stage three simulations accounted for a couple of minorities in the same network (population).

It can be showed that, as expected, similar configurations led to trivial outcomes, namely, when both of the committed minority configurations are compact (or both sparse), if both are fixed at +1, then $\xi_{M}$ = 1, if both are fixed at −1, then $\xi_{M}$ = −1 and if one is +1 and the other −1, then $\xi_{M}$ = 0.

Interesting outcomes appeared when sparse config versus compact config were simulated. In particular, when the same value was fixed for both configurations, the global value of course confirmed the minority value. On the other hand, when different configurations challenge each other, the results were the following:

Sparse config = −1 versus compact config = +1 led to ${\bar{\xi }}_{eq}$ = −0.56 ± 0.02 hence $\xi_{M}$ = −1;
Sparse config = +1 versus compact config = −1 led to ${\bar{\xi }}_{eq}$ = 0.59 ± 0.03 hence $\xi_{M}$ = +1.

In brief, the sparse config somehow “dominated” the compact configuration, showing a clearly stronger influence on the population dynamics.

It could be argued that ${\bar{\xi }}_{eq}$ seems to be “weak” because although it is in the proper range (e.g., we have ${\bar{\xi }}_{eq}\;$*=* −0.56 ± 0.02, so ${\bar{\xi }}_{eq}<-0.33$, hence $\xi_{M}$ = −1) and with a small SD, it is not close to the reference value −1. In this regard, it should be considered that we are simulating with four elements only in each committed minority group, corresponding to only 1% of the population. It can be easily shown that when slightly increasing elements, ${\bar{\xi }}_{eq}$ can increase as well. For instance, when performing the same simulations with the committed minority group at 3%, we have:

Sparse config = −1 versus compact config = +1 led to ${\bar{\xi }}_{eq}\;$= −0.65 ± 0.02;
Sparse config = +1 versus compact config = −1 led to ${\bar{\xi }}_{eq}\;$= 0.60 ± 0.03.

Therefore, without a loss of generality, we should keep the 1% minority as a kind of standard minimum, in order to show properties becoming stronger and just slightly increasing the number of individuals within the committed minorities.

### Appendix A.2. (b) Simulations at Supercriticality

When the lattice is in a supercritical condition (e.g., *K* = 2.7), it “collapses” down to the “+1” or “−1” global state (i.e., to $\xi_{eq}\;$> 0.95 or, respectively, $\xi_{eq}\;$< −0.95). The “decision” depends on the lattice’s initial condition and not on possible committed minorities, as we can show through two sets of five trials.

In the first part (first five trials), we set any element of the lattice with initial values at +1 and a compact committed minority at −1. We found a final $\xi_{eq}\;$= 0.96 ± 2.89 × 10^−5^ (m ± SD).

Secondly, (second five trials), setting the initial values at −1 and a sparse committed minority at +1, we found $\xi_{eq}\;$= −0.96 ± 4.90 × 10^−4^, showing that, in supercritical conditions, the lattice initial condition is the main point, stronger than any committed minority. Hence, the majority of the nodes dominate in any committed minority. In particular, when the lattice is in a supercritical condition, it “collapses” down to the “+1” or “−1” global state according to the state of the majority in the initial condition. In order to further verify this, we performed 10 trials with an initial lattice value at +1 for 55% of the nodes (random starting position). We found $\xi_{eq}$ = +0.97 ± 3.31 × 10^−2^ and that 100% of them went to $\xi_{M}$ = +1.

On the other hand, we then performed 10 trials with an initial lattice value at −1 for 55% of the nodes (random starting position). We found $\xi_{eq\;}$= −0.98 ± 1.28 × 10^−2^ and that 100% of them went to $\xi_{M}$ = −1.

## Appendix B. The DMM Starting Point Dependence at Criticality

We simulated two different scenarios at criticality:

Scenario A: a committed minority (−1) in a sea of +1 elements (all of the elements not in the committed minority are +1);

Scenario B: a committed minority (−1) in a sea of −1 elements (all of the elements not in the committed minority are −1).

Simulation conditions and parameters are the same as in Appendix A unless otherwise specified. In particular, the *K* parameter is *K* = 1.66.

We analyze the iteration dynamics in the first 10^4^ iterations (we performed 10 simulations for each scenario). Descriptive statistics results are reported in Table A1. In the second column (named “% of −1”) the mean percentage of iterations with $\xi_{eq}\;$< 0 (in a total of 10^4^) is reported. It is clearly shown that, considering the first 10,000 iterations, the probability of having the majority with a −1 status is very much larger in Scenario B (namely, starting from a fully −1 layout).

**Table A1:** Results of simulation for two scenarios at criticality. The second column reports the mean percentage of iterations with $\xi_{eq\;}\;$< 0, the third column reports the standard deviation (SD) over the 10 trials and Min and Max columns are the minimum and the maximum resulting from the 10 trials.

Scenarios	% of −1	SD	Min	Max
Scenario A	68.95	21.09	38.39	88.36
Scenario B	98.78	3.52	88.80	100

A qualitative comparison reporting an example of Scenario A and Scenario B (first 10^4^ iterations) can be seen in Figure A1.

This 10 + 10 set of simulations for Scenario A and B clearly shows that, at criticality, different starting layouts lead to different outcomes, at least in the first iterations: the final outcome depends on the starting layout.
